# Supplementary figures and images for: Phenotypic impact of individual conserved neuronal microexons and their master regulators in zebrafish
Source: eLife. 2025 Nov 18;13:RP104275. doi: 10.7554/eLife.104275 (PMC12626424; doi:10.7554/eLife.104275)

top panel

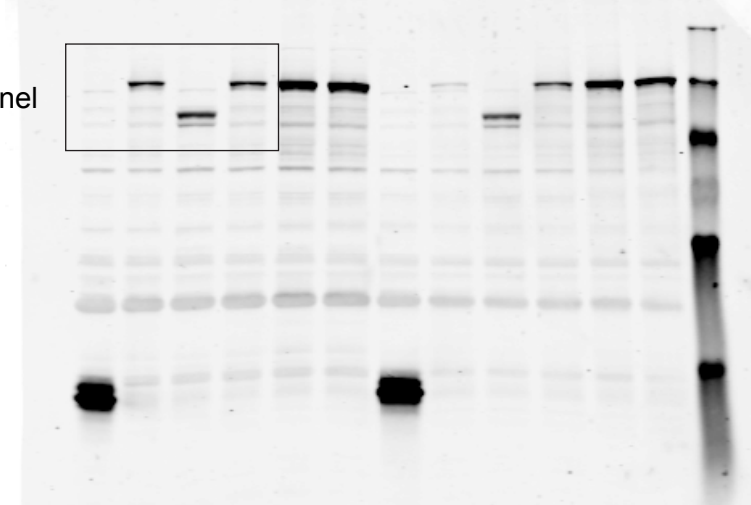

bottom panel

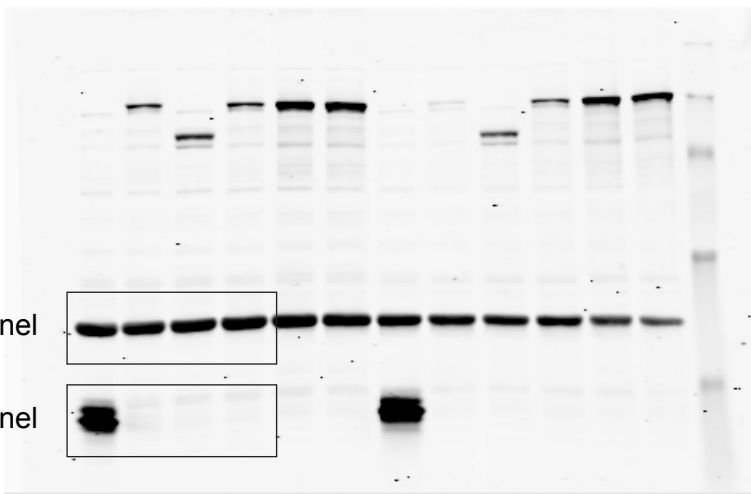

middle panel

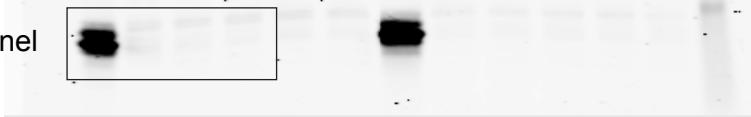

Supplement: Figure 3—source data 1. [file elife-104275-fig3-data1.pdf]
