# Supplementary material for: Phenotypic impact of individual conserved neuronal microexons and their master regulators in zebrafish
Source: eLife. 2025 Nov 18;13:RP104275. doi: 10.7554/eLife.104275 (PMC12626424; doi:10.7554/eLife.104275)
Supplement: MDAR checklist [file elife-104275-mdarchecklist1.pdf]

Westbrook Centre
Milton Road
Cambridge CB4 1YG, UK

P 01223 855340
W elifesciences.org
T @elife

# Materials Design Analysis Reporting (MDAR) Checklist for Authors

The MDAR framework establishes a minimum set of requirements in transparent reporting mainly applicable to studies in the life sciences.

*eLife* asks authors to **provide detailed information within their article** to facilitate the interpretation and replication of their work. Authors can also upload supporting materials to comply with relevant reporting guidelines for health-related research (see EQUATOR Network), life science research (see the BioSharing Information Resource), or animal research (see the ARRIVE Guidelines and the STRANGE Framework; for details, see *eLife*'s Journal Policies). Where applicable, authors should refer to any relevant reporting standards materials in this form.

For all that apply, please note **where in the article** the information is provided. Please note that we also collect information about data availability and ethics in the submission form.

## Materials:

| Newly created materials | Indicate where provided: section/figure legend | N/A |
|---|---|---|
| - Zebrafish lines. | Generation of microexon deletion lines using CRISPR-Cas9 and Key resource table | |

| Antibodies | Indicate where provided: section/figure legend | N/A |
|---|---|---|
| Commercial antibodies | Key resource table | N/A |

| DNA and RNA sequences | Indicate where provided: section/figure legend | N/A |
|---|---|---|
| Primers for RT-PCR validations and CRISPR gRNAS | Supplementary files 3 and 4, Key resource table | N/A |

| Cell materials | Indicate where provided: section/figure legend | N/A |
|---|---|---|
| Cos-7 | Figure 3I and Key resource table. | |
| Primary neuronal cultures of zebrafish | Figure 3 and supplements. | N/A |

| Experimental animals | Indicate where provided: section/figure legend | N/A |
|---|---|---|
| All zebrafish mutant lines were generated using the Tg(HuC:GFP) line (Park, et al. 2000) as background. To preserve colony health and maintain genetic diversity, an AB wild-type line was regularly incorporated. Fish were outcrossed with AB individuals every two generations to prevent inbreeding. | Provided by Elisa Martí's laboratory at the Institute of Molecular Biology of Barcelona (IBMB) | |
| | | N/A |

| Plants and microbes | Indicate where provided: section/figure legend | N/A |
|---|---|---|
| | | N/A |
| | | N/A |

| Human research participants | Indicate where provided: section/figure legend) or state if these demographics were not collected | N/A |
|---|---|---|
| | | N/A |

## Design:

| Study protocol | Indicate where provided: section/figure legend | N/A |
|---|---|---|
| | | N/A |

| Laboratory protocol | Indicate where provided: section/figure legend | N/A |
|---|---|---|
| | | N/A |

| Experimental study design (statistics details) * | | |
|---|---|---|
| **For in vivo studies: State whether and how the following have been done** | **Indicate where provided: section/figure legend. If it could have been done, but was not, write "not done"** | **N/A** |
| Various statistical details and considerations | See each Methods section and "Considerations regarding statistical analyses" | |
| | | |
| | | |
| | | |

| Sample definition and in-laboratory replication | Indicate where provided: section/figure legend | N/A |
|---|---|---|
| Replicates are stated in each figure and throughout the MS. | Throughout the manuscript | |

| | | |
|---|---|---|
| Define whether data describe technical or biological replicates. | Data describe biological replicates (individual cells or animals). | |

| **Ethics** | **Indicate where provided: section/submission form** | **N/A** |
|---|---|---|
| | | N/A |
| Experiments with zebrafish | Fish procedures were approved by the Institutional Animal Care and Use Ethic Committee (PRBB–IACUEC) | |
| Studies involving specimen and field samples: State if relevant permits obtained, provide details of authority approving study; if none were required, explain why. | | N/A |

| **Dual Use Research of Concern (DURC)** | **Indicate where provided: section/submission form** | **N/A** |
|---|---|---|
| If study is subject to dual use research of concern regulations, state the authority granting approval and reference number for the regulatory approval. | | N/A |

## Analysis:

| Attrition | Indicate where provided: section/figure legend | N/A |
|---|---|---|
| | | |

| Statistics | Indicate where provided: section/figure legend | N/A |
|---|---|---|
| Statistical tests are described through the MS | See methods section. | |

| Data availability | Indicate where provided: section/submission form | N/A |
|---|---|---|
| Data availability statement is included both in the MS and in the submission system. | See section in the manuscript. | |
| | | N/A |
| | | |

| Code availability | Indicate where provided: section/figure legend | N/A |
|---|---|---|
| Relevant software are provide in Source code 1.Rmd (along with the necessary raw data) or in https://github.com/vastgroup/mic-social-behavior-analysis. | Code is reported in the availability statement | |
| | | N/A |

| | | |
|---|---|---|
| | | N/A |

## Reporting:

The MDAR framework recommends adoption of discipline-specific guidelines, established and endorsed through community initiatives.

| Adherence to community standards | Indicate where provided: section/figure legend | N/A |
|---|---|---|
| | | N/A |

---

* We provide the following guidance regarding transparent reporting and statistics; we also refer authors to [Ten common statistical mistakes to watch out for when writing or reviewing a manuscript](#).

**Sample-size estimation**

- You should state whether an appropriate sample size was computed when the study was being designed
- You should state the statistical method of sample size computation and any required assumptions
- If no explicit power analysis was used, you should describe how you decided what sample (replicate) size (number) to use

**Replicates**

- You should report how often each experiment was performed
- You should include a definition of biological versus technical replication
- The data obtained should be provided and sufficient information should be provided to indicate the number of independent biological and/or technical replicates
- If you encountered any outliers, you should describe how these were handled
- Criteria for exclusion/inclusion of data should be clearly stated
- High-throughput sequence data should be uploaded before submission, with a private link for reviewers provided (these are available from both GEO and ArrayExpress)

**Statistical reporting**

- Statistical analysis methods should be described and justified
- Raw data should be presented in figures whenever informative to do so (typically when N per group is less than 10)
- For each experiment, you should identify the statistical tests used, exact values of N, definitions of center, methods of multiple test correction, and dispersion and precision measures (e.g., mean, median, SD, SEM, confidence intervals; and, for the major substantive results, a measure of effect size (e.g., Pearson's r, Cohen's d)
- Report exact p-values wherever possible alongside the summary statistics and 95% confidence intervals. These should be reported for all key questions and not only when the p-value is less than 0.05.

**Group allocation**

- Indicate how samples were allocated into experimental groups (in the case of clinical studies,

please specify allocation to treatment method); if randomization was used, please also state if restricted randomization was applied

- Indicate if masking was used during group allocation, data collection and/or data analysis
